# Draft Genome Sequence of *Streptomyces* sp. Strain I05A-00742, Isolated in Shangri-La, China

**DOI:** 10.1128/MRA.00521-20

**Published:** 2020-06-25

**Authors:** Xingxing Li, Wei He, Hongmin Sun, Yuanyuan Shi, Xiumin Zhang, Yunying Xie, Li Wang, Bin Hong

**Affiliations:** a NHC Key Laboratory of Biotechnology of Antibiotics, Institute of Medicinal Biotechnology, Chinese Academy of Medical Sciences & Peking Union Medical College, Beijing, China; b CAMS Key Laboratory of Synthetic Biology for Drug Innovation, Institute of Medicinal Biotechnology, Chinese Academy of Medical Sciences & Peking Union Medical College, Beijing, China; Broad Institute

## Abstract

*Streptomyces* sp. strain I05A-00742 was isolated from a soil sample in Napahai in Shangri-La, Yunnan Province, China. Here, we report the draft genome sequence of *Streptomyces* sp. I05A-00742, which consists of an assembly size of 7,129,054 bp in 105 scaffolds with a G+C content of 72.43%.

## ANNOUNCEMENT

*Streptomyces* sp. strain I05A-00742 is a Gram-positive bacterium that was isolated from a soil sample in Napahai in Shangri-La, Diqing Tibetan Autonomous Prefecture, Yunnan Province, China. Piericidin A1 has been shown to have many biological activities, including insecticidal (1), antifungal (2), and antitumor (3) activities. Three piericidin A1 biosynthetic gene clusters have been characterized in Streptomyces piomogenus variant Hangzhouwanensis (4), *Streptomyces* sp. strain SCSIO 03032 (5), and Streptomyces philanthi bv. triangulum (2) and display similar genetic organization. Here, we report the draft genome sequence of *Streptomyces* sp. I05A-00742, a potential piericidin producer revealed by its genome sequence.

For DNA extraction, *Streptomyces* sp. I05A-00742 was grown for 1 week on mannitol soya flour (MS) agar (6) at 28°C and then transferred to ISP medium 2 (0.4% yeast extract, 10% malt extract, and 0.4% glucose [pH 7.2]) at 28°C for 48 h. The genomic DNA was isolated using the Wizard genomic DNA purification kit (Promega, USA) and assessed using a NanoDrop 2000 spectrophotometer and agarose electrophoresis. The genomic DNA library was generated using the NEXTflex rapid kit (Bioscientific). The draft genome sequence of *Streptomyces* sp. I05A-00742 was obtained on a second-generation sequencing platform, Illumina HiSeq X Ten platform (2 × 150-bp paired-end reads), resulting in 1,436,443,672 bp of raw data (9,512,872 paired-end reads with a 415-bp average insert size and a sequencing depth of 200×). After sequencing, adapters were trimmed, and low-quality reads were filtered out using SeqPrep (https://github.com/jstjohn/SeqPrep) and Trimmomatic v0.36 (7) with default settings. A total of 1,340,847,504 bp of clean data (9,050,014 reads) was obtained. The draft genome sequence was directly assembled into 180 contigs (7,128,881 bp; *N*_50_, 90,220 bp) and then composed into 105 scaffolds (7,129,054 bp; *N*_50_, 166,849 bp; G+C content, 72.43%) by SOAPdenovo v2.04 (parameter setting, kmer 21 to 41) (8) and GapCloser v1.12 (8). There are 590 tandem repeat sequences (88,920 bp), which make up 1.44% of the genome, found by Tandem Repeats Finder v4.07b (9) (parameter settings were match = 2, mismatch = 7, delta = 7, PM = 80, PI = 10, minscore = 80, and maxperiod = 500).

In the draft genome sequence of *Streptomyces* sp. I05A-00742, 33 putative secondary metabolite biosynthetic gene clusters were found using antiSMASH v5.1.2 (default setting) (10). Of these clusters, the putative piericidin A1 biosynthetic gene cluster is located in scaffold 13, and its gene content and amino acid sequences show high similarity with the reported piericidin A1 clusters (2, 4, 5). In addition, antiSMASH revealed some gene clusters related to the biosynthesis of analogues of ketomemicin, qinichelins, and citrulassin, as well as a number of clusters which are not highly conserved with any known cluster. The genome sequence of *Streptomyces* sp. I05A-00742 may facilitate a better understanding of the biosynthesis of piericidins in different producing strains and provide insights into its metabolic potential for novel compounds.

### Data availability.

This draft genome sequence of *Streptomyces* sp. strain I05A-00742 has been deposited at DDBJ/ENA/GenBank under the BioProject number PRJNA629417, BioSample number SAMN14775352, and GenBank accession number JABETO000000000. The version described in this paper is the first version, JABETO010000000. Clean sequence reads have been deposited in the Sequence Read Archive (SRA) database under the accession number SRR11648388. *Streptomyces* sp. I05A-00742 was deposited at the China Pharmaceutical Culture Collection (CPCC 205389).
